# Prof. Mahla’s Report on the Chicago River

**Published:** 1862-10

**Authors:** 


### CHICAGO MEDICAL EXAMINER.

N. S. DAVIS, M.D., EDITOR.

VOL. III. OCTOBER, 1862. NO. 10.

### Original Contributions.

ARTICLE XXXI.

### PROF. MAHLA'S REPORT ON THE CHICAGO RIVER.

*To the Committee of Aldermen, appointed for the Investigation of the Sources of Impurity in Chicago River:*

GENTLEMEN:—I have the honor to present to you herewith the result of a chemical investigation, instituted at your request, with a view to trace the impurity of the Chicago River to its cause or causes. Difficult as this task is by itself, its complication has been considerably increased by certain circumstances beyond our control. Nevertheless, I trust that certain facts, exhibited by the accompanying table, will prove satisfactory to the members of the Common Council, and the public in general, where to locate the source of these impurities.

The odorous bodies which proceed from the Chicago River water are products of the decay and decomposition of animal and vegetable matter. Amongst them we recognize ammonia, sulphuretted and phosphuretted hydrogen, light carburetted hydrogen (marsh gas,) and scores of others, which are either of minor importance, or are summed up under the mysterious name "miasmatic effluvia."

The above-named combinations are produced in variable proportions, ammonia being the most prominent and important amongst them. This substance is generated always when animal or vegetable azotized materials are undergoing putrefaction or decay. It is a well-known fact that animal refuse matters are rich in nitrogen, and so is the vegetable refuse produced in distilleries, and known there under the peculiar term of "slop" or "swill." It is evident, therefore, that the slaughter and feeding houses, the tanneries, distilleries, and glue factories, if throwing such refuse matter into the River, admix to it ingredients that are rapidly redissolving their constituents into simpler compounds, *one of which is ammonia.* It need hardly be remarked here that the same body is contained in sewage, and that it is generated in considerable quantities by the dry distillation of coal. In so far now as this compound, ammonia, admits of a very accurate determination, I directed my attention entirely to it, disregarding fully the inorganic mineral constituents, because they cannot, under any circumstances, take an active part in the production of odorous bodies.

The small amount of current existing in the Chicago River is controlled almost entirely by the influence of winds. It is a fact well known to any one who pays attention to it, that a north or northeast wind makes the water rise in our River, whilst a southern or southeastern makes it fall. With other words, a northerly wind produces an upward, while a south wind causes a downward current. An east wind has rather the former effect, while a west wind drives it more or less outwards. If the wind be not very strong, all these movements are but slight and slow, and hence it is true that the Chicago River is more or less stagnant, in so far as the supply of fresh water from its origin is not very abundant. In consequence of these facts, any impurities emptied into the River remain in it for considerable time, or are, under a favorable wind, slowly driven into the Lake. The decomposition of animal and vegetable substances begins almost at the very spot where they mix with the River, or, if odorous principles in free state are admixed to it, they are found not far from their place of production in *larger* proportion than in others.

These considerations evidently point out the course of investigation. An accurate determination of the quantity of ammonia in different parts of the River must fairly indicate a rapid increase of deteriorating substances, and must lead to the discovery of the parties by whom nuisances are produced. It was, however, absolutely necessary, in order to obtain reliable results, to collect the water only when the current of the River was downward, and in so far as this is controlled by the direction of winds, this had to be borne in mind.

The impossibility of collecting the different specimens of water for the purpose of a chemical analysis, at once, is obvious. The materials which are producing ammonia are subject to such rapid changes, that it was deemed advisable not to collect more specimens of water than could be analyzed within a reasonable time. To establish, however, a ratio between the various samples, I collected with every new series a specimen of water from a locality which had been analyzed at a previous time. Every rain had a certain bearing on the condition of the water—a fact which is fully demonstrated by the data given in the subjoined table. A heavy rain had made its appearance just one day before the first specimens of water were collected. As it takes, however, four or five days before the rain water flows into the River, it is easily intelligible that an amelioration in the condition of the water can be observed only after the lapse of such a time. The inference referred to can be appreciated by glancing at columns II, III, IV, V, and comparing the results obtained from the specimens 1, 2, 3, 4, 5 with 7, 8, 9, etc., also 10 with 17.

As a general thing, it was found that the specimens of water taken from the South Branch were purer than those from the North Branch of the River, (compare 17 with 18 and 19; also, 27 and 28 with 29 and 30, and 31.) This is undoubtedly due to the fresh water with which that Branch is supplied from the Desplaines River, but still more so from the Canal. By descending the River, it is found that the first rapid increase in the quantity of ammonia takes place between Van Buren and Madison streets, or rather in the vicinity of Monroe street, (compare 8 with 9; also, 12 with 13, 14, 15, and 16.) The amount of ammonia increases slowly by going down into the main River, and reaches its maximum between Rush and Clark streets. The first specimen of water from the North Branch was taken far above all factories, at a locality where it would be supposed that the River water contained both organic constituents, and especially ammonia in no larger proportions than they would be found in ordinary River water. (See 18.)

Descending the North Branch from that place, it can be seen that the deteriorating materials commence increasing at Clybourne avenue, (below Nickerson's and above Wicker & Co.'s; compare 18 and 19.) The condition of the River becomes, however, rapidly worse between Clybourne and North avenues, (compare 19 with 20.) The same is observed between the latter place, Division street, and Chicago avenue, (compare 29, 30, and 31.) From there, however, to Indiana street, the ammonia augments in no greater proportion than would be attributed to natural causes, (compare 21 and 22.) A river being the drain of a country or a town through which it flows, must of course dissolve all the impurities which enter it in its course.

These same observations can be made by every layman, simply by testing for sulphuretted hydrogen. This combination is not contained in free state in the River water, but united with ammonia. Lead-paper, when held over the surface of the River water contained in a half-gallon bottle, is not affected, provided the specimen be not taken from the immediate neighborhood of a sewer. It is discolored, however, rapidly, if a few drachms of concentrated sulphuric acid are added. This substance not only liberates that body, but heats the River water sufficiently to disengage the gas. The rapidity with which the discoloration is effected, and the darkness of the paper, will give at least an idea as to the impregnation of the water with that body. It is obvious, however, that such a proceeding cannot serve for any *accurate* determinations. The fact now that the North Branch of the River is lined, above Chicago avenue, with distilleries, tanneries, slaughter-houses, stables, and hog-pens, (in some of which more than 1000 head are kept,) explains the results obtained by chemical analysis. The urine, mixed with the *fæces* of the pigs and cattle kept in those stables, is poured into the

River. Occasionally the slop of the distilleries runs over and is added, with the blood of the slaughter-houses, to the decaying substances. Under such circumstances, what else could be expected than the commencement of a rapid decomposition, started by the action of numerous bodies, all of which are already engaged in the same process? And is it to be wondered that the water of the North Branch is *almost as impure as that taken from one of our most important sewers?* (Compare 29, 30, 31, with 34, 35, 36.

Another observation which was made in the course of these investigations is the following:

Copious masses of gas rise to the surface of the river water between Clybourne avenue and Division street, especially, however, in the neighborhood of the Chicago Distillery Co. (Rawson & Streeter.) This takes place in such proportions that—to use the language of the people living there—the River boils. This gas was found to be a mixture of carbonic acid and light carburetted hydrogen (marsh-gas.)

The water of the North Branch is always darker than that of the South Branch of the River, provided the River be in its natural condition, undisturbed by the pumping operations of the Illinois and Michigan Canal at Bridgeport, or cleaned out by heavy rains. This difference in color was especially remarkable during the last eight days, and reached such a degree that the first one appeared almost black when compared with the other. A great deal of curiosity has been manifested by the public as to the probable cause of this black color. In my opinion, it is a natural consequence of the decay of vegetable substances, and readily intelligible. The slop of distilleries consists of a variety of substances, some of which are of great value for fattening cattle, and are used by several parties for that purpose. The cellulose, however, which is an important item of the constituents of slop, is indigestible, and passes off unaltered with the *fæces.* This cellulose now is the material, which, by its decay, produces, at a certain period, the black body, which, when suspended in the water, imparts to the latter its remarkable black color. In order to explain my views, I am forced here to call an analogous fact to remembrance—the formation of coal. I do not mean that coal formation which took place in the earliest periods of the earth, but one which is still progressing. KNAPP says, in this respect, in his excellent work on chemical technology: "It would be going too far, if we would deny the continuous formation of coal, even at the present time. The large rivers of the American continent carry often numbers of trees away, which become heavier the more they are soaked with water, and sink finally to the ground. There they are covered with mud, and undergo a slow process of decay. Such trees are converted, after some time, into brown coal. A similar process is the formation of peat, and, I hold, that the formation of the black body in our River is an analogous fact. Let us call this product, formed from cellulose, ulminic acid; or let us denominate it otherwise. The fact is, it is a derivitive which is richer in carbon than the original cellulose, and formed from it. If we compare the composition of cellulose with that of brown coal or ulminic acid, we will readily find that the latter two are richer in carbon than the first; and it is now generally assumed that this change is produced by the elimination of oxygen and hydrogen, with a little carbon in the form of carbonic acid, marsh-gas and water.

Cellulose = $C^{24}\ H^{20}\ O^{20}$

If we subtract: 8 equiv. water, = $H^8\ O^8$

5 equiv. carb. acid, $C^5\ O^{10}$

5 equiv. marsh gas, $C^5\ H^{10}$ } = $C^{10}\ H^{18}\ O^{18}$

we obtain in the following, viz.:— $C^{14}\ H^2\ O^2$

the formula of a body that is very similar in its composition to certain varieties of brown coal. Or,

1 equiv. cellulose, = $C^{24}\ H^{20}\ O^{20}$

decomposes into

1 equiv. ulminic acid = $C^{20}\ H^6\ O^6$

2 equiv. carbonic acid = $C^2\ O^4$

2 equiv. carburetted hydrog. = $C^2\ H^4$

10 equiv. water = $H^{10}\ O^{10}$ } = $C^{24}\ H^{20}\ O^{20}$

This argument seems to be substantiated by the observation that both carbonic acid and marsh-gas are evolved in such copious masses from the bottom of the River, (as stated above,) and that the black color begins just below the still-houses and stables.

Thus it appears that the largest portion of the River nuisance is generated on the North Branch, and it must be borne in mind that *not a single sewer* empties into that part of the River.

It cannot be disputed, however, that the sewers themselves give rise to a certain portion of the odorous effluvia, and will do this in a still greater measure in the course of time, (a thing which was foreseen by our able Chief Engineer, Mr. E. S. CHEESEBROUGH, and mentioned by him in his report as early as 1855.) But considering that the sewers, which are of importance, are emptying their liquids into the Main Branch of the River, so that the sewage has but a short distance to flow until it mixes with Lake water, and is largely diluted with it. I do not believe that their effects are for the present specially noxious.

It may be stated here that all the sewers south of Madison street, with the exception of a single one, are of not much importance, and that the sewage contained in them is but little richer in ammonia than the River water itself. The single sewer, however, to which I have made reference is the Monroe street sewer. The liquid contained in it is very rich in ammonia, (compare 32); contains coaltar-creosote (carbolic acid), and exhales copious quantities of sulphuretted hydrogen; a lead-paper held in a distance of four or five feet from the surface of the water is blackened almost immediately, provided that the current has a riverward direction. The discoloration of lead-paper is effected in a much slighter degree by the sewage of other sewers.

Admixed to the liquid discharged by the Monroe street sewer are tarry substances, which have accumulated in the River to such an extent that its whole bed seems to be covered with it, for every steam tug wheeling up the water makes a portion of tar rise to the surface. The presence of these tarry substances can be traced from Adams street to Rush street bridge. Sulphuric ether agitated with the water extracts them readily; they may be separated from the ethereal solution by a subsequent spontaneous evaporation.

It appears from the tables, that the sewage contained in the Rush street sewer is less charged with ammonia than other sewers, (compare 33, 34, 35, 36.) This may look strange to some, as it is known that the greatest number of privies are connected with it. The water in this sewer is in a condition perfectly different from others. It is hot, (heated by the condensing waters of the pumping works and breweries,) and hence less apt to hold gaseous bodies in solution. Ammonia and its associates of miasmatic effluvia are, therefore, sent into the air as soon as generated. Hence this sewer becomes often very offensive to the inhabitants of the street, much more so than Randolph street sewer to the inhabitants of that street.

From the above, it may be seen that, 1st. The greatest portion of the impurities which give rise to putrid effluvia enter into the North Branch of the Chicago River; that the urine, fæces, etc., fed on the swill of the still-houses, by being allowed to flow into this Branch, are the most direct causes of noxious exhalations; that certain distilleries, in allowing portions of the swill to run into the River, contribute also their share to them.

2. The Monroe street sewer discharges into the River a liquid which contains odorous substances that are usually produced in gas factories, which are more disagreeable than noxious.

3. The city sewers alone are not yet of sufficient importance to create serious trouble, but will do so undoubtedly in the course of time.

4. The slaughter and packing-houses on the South Branch, cannot be blamed *at present*, (compare 3, 4, 5,)—a conclusion which may be inferred from the fact that they are not, and have not, been working for several months previous to the commencement of this investigation. The question whether they do throw waste materials into the River, when working, can be settled only in spring-time, after the River ice has given way.

It is undoubtedly a matter of great importance how the existing nuisances can be abated. I cannot omit, however, to make here the remark, that not all the odorous effluvia are sent forth by the Chicago River. The packing and slaughter-house owners are at present not doing anything which is objectionable or deteriorating our River water. But, if my information is correct, they leave large quantities of refuse matter in certain places near by, where they undergo putrefaction. The odor in the vicinity of such establishments is unbearable; and, I ask now, is the stench by which Bridgeport is infested, and which can be observed during a favorable wind, even in our city business streets, less objectionable because it proceeds directly from the soil on which the animal refuse is thrown, and does not emanate from the River water? Are the gaseous bodies generated by the putrefaction process less noxious in the first than in the latter case?

I should propose that these men, instead of throwing a valuable azotized compound away, should use it up *for the production of saltpetre*, which is an article commanding invariably a certain price, that would not only cover expenses, but secure a handsome profit. The same might be done by the owners of the stables and piggeries with the urine and manure. The gas factory, however, instead of pouring the wash-water into the River, could use them for the *production of ammoniacal salts*, a thing which is done in most of the European cities, with a great deal of advantage. By doing so, the offensive sulphuretted hydrogen could be got rid of in a more complete manner than by using dry lime purifiers.

In conclusion, I wish to state that the determinations of ammonia were made by volumetric analysis, principally following Mr. BOUSSINGAULT'S plan, and adopted by him in the determination of that body in rain, snow, river, and spring waters, (*Annales de chimie et Phys.* xxxix, page 257; also, *Mohr's Hand-Book der Titrir methode*, page 77.)

| No. of specimen of water. | Date of Collection. | | Where Collected. SOUTH BRANCH. | I. Wind. | II. Rain, inch's. | III. Organic constituents. | IV. Ammonia in 100.000 p'rts. | V. Ratio of Ammonia in the difn't spe'ns. |
|---|---|---|---|---|---|---|---|---|
| 1 | July | 22 | Above Wahl's glue factory, | s | ...... | 0.56726 | ............ | ............ |
| 2 | " | 23 | Archer road bridge, | w | ...... | 0.51343 | ............ | ............ |
| 3 | " | 23 | Opposite Hancock's packing house, | w | ...... | 0.40944 | 0.216720 | 998 |
| 4 | " | 23 | Halsted street bridge, | w | ...... | 0.41474 | 0.217152 | 1000 |
| 5 | " | 23 | Twelfth street bridge, | w | ...... | 0.41804 | 0.282168 | 1299 |
| 6 | " | 23 | Clark street bridge, | n w | ...... | 0.70440 | ............ | ...... |
| 7 | " | 27 | Twelfth street bridge, | e s e | ...... | 0.36202 | 0.108180 | 1299 |
| 8 | " | 27 | Polk street bridge, | e s e | ...... | 0.36632 | 0.125280 | 1504 |
| 9 | " | 27 | Madison street bridge, | e s e | ...... | 0.38071 | 0.223200 | 2680 |
| 10 | " | 29 | Halsted street bridge, | e s e | ...... | 0.36704 | 0.146844 | .1000 |
| 11 | " | 29 | Twelfth street bridge, | e s e | ...... | 0.37208 | 0.190800 | 1299 |
| 12 | " | 29 | Van Buren street bridge, | e s e | ...... | 0.39693 | 0.233460 | 1589 |
| 13 | " | 29 | Opposite Monroe street, middle of River, | e s e | ...... | 0.40283 | 0.276948 | 1885 |
| 14 | Augt. | 1 | Twelfth street bridge, | w | ...... | 0.38622 | 0.244764 | 1299 |
| 15 | " | 1 | Dock, just south of Monroe, | w | ...... | 0.43600 | 0.417384 | 2211 |
| 16 | " | 1 | Randolph street bridge, | w | ...... | 0.57538 | 0.419868 | 2228 |
| | " | 5 | ............ | ...... | 7 | ............ | ............ | ............ |

| No. of specimen of water. | Date of collection. | | Where Collected. NORTH BRANCH. | I. Wind. | II. Rain inches. | III. Organic constituents. | IV. Ammonia in 100.000 parts. | V. Ratio of ammonia in difer'nt specm's. |
|---|---|---|---|---|---|---|---|---|
| 17 | Augt. | 8 | Halsted street bridge, South Branch, for comparison, | s | ...... | 0.04805 | 0.10006 | 1000 |
| 18 | " | 8 | Bridge opp. Faber's bone black factory, almost a mile n. of RR. crossing & Nicker's. | s | ...... | 0.05010 | 0.09945 | 993 |
| 19 | " | 8 | Clybourne ave. bridge, below Nickerson's, and above Wicker & Co.'s distillery, | s | ...... | 0.06571 | 0.11475 | 1146 |
| 20 | " | 8 | North avenue, below Rawson & Streeter's, | s | ...... | 0.06982 | 0.19125 | 1911 |
| 21 | " | 8 | Chicago avenue bridge, | s | ...... | 0.08338 | 0.23290 | 2327 |
| 22 | " | 8 | Indiana street bridge, | s | ...... | 0.08625 | .0.25583 | 2556 |
| | " | 9 | ............ | ...... | 1¾ | ............ | ............ | ...... |
| 23 | " | 10 | Above foot of Franklin street,. | s w | ...... | 0.055037 | 0.25670 | ...... |
| 24 | " | 10 | Below Clark street bridge, | s w | ...... | 0.066538 | 0.30600 | ...... |
| 25 | " | 10 | Below Rush street bridge, | s w | ...... | 0.066948 | 0.34425 | ...... |
| 26 | " | 22 | Halsted street bridge, | s w | ...... | ............ | 0.1728 | 1000 |
| 27 | " | 22 | Van Buren street bridge, | s w | ...... | ............ | 0.2754 | 1593 |
| 28 | " | 22 | Madison street bridge, | s w | ...... | ............ | 0.3876 | 2254 |
| 29 | " | 22 | Chicage avenue bridge, | s w | ...... | ............ | 1.3243 | 7663 |
| 30 | " | 22 | Division street, | s w | ...... | ............ | 1.3022 | 7535 |
| 31 | " | 22 | North avenue bridge, | s w | ...... | ............ | 1.1526 | 6670 |
| 32 | " | 22 | Monroe street bridge, | s w | ...... | ............ | 2.7200 | 15740 |
| 33 | " | 22 | Randolph street sewer of South side, | s w | ...... | ............ | 2.6112 | 15111 |
| 34 | " | 22 | Randolph street sewer of West side, | s w | ...... | ............ | 1.0676 | 6178 |
| 35 | " | 22 | Clark street sewer of North side, | s w | ...... | ............ | 1.4502 | 8392 |
| 36 | " | 22 | Rush street sewer of North side, | s w | ...... | ............ | 0.7199 | 4166 |

### EXPLANATION OF THE TABLE.

Column I. shows the direction of the wind at the time of collection.

Column II. shows the quantity of rain fallen within 24 hours on the square foot of surface—expressed in inches.

Column III. exhibits the proportion of organic matter in several specimens of water.

Column IV. gives the actual quantity of ammonia in 100.000 parts.

Column V. exhibits the ratio of ammonia in the different samples of water. In comparing the various specimens, I had to assume one of them as unity, which I did by giving to the water collected at Halsted street bridge the round cipher 1000. The fractional numbers exhibited in column IV. were assumed as whole numbers, and all of them reduced to the standard of Halsted street—1000.

The titre solutions used by me were, however, only one-tenth of the normal strength, thus making the test more delicate.

The organic matter was determined by normal solution of mineral chameleon, under addition of some cont. sulphuric acid. The number of cubic centimeters used were reduced to oxalic acid. It was assumed that the reaction was ended when the rose color lasted for a period of five minutes. Though this method is very dispatching, and for the present purpose immensely preferable to the old mode of incinerating the dry residue of a given quantity of water, it is evident that the direct quantities of organic matter are not found by it. *All that is found is the proportion* of organic constituents in various specimens of water. The foregoing results are all that can be ascertained by an investigation at this season of the year. The additional effects produced upon the water by the refuse of slaughter and packing-houses can only be known in spring-time.

